# Assessment of artificial ventilation in Salvadorian public hospitals

**DOI:** 10.1186/cc9936

**Published:** 2011-03-11

**Authors:** JF Granados, PE Sobenes, MT Bertoli

**Affiliations:** 1 Dr Jose Matias Delgado University, Antiguo Cuscatlan, El Salvador

## Introduction

Artificial ventilation (AV) is an essential resource that is not always available at Salvadorian public hospitals. Approximately 40% of hospital-related deaths may have required AV [1]. The main indication for AV is respiratory insufficiency requiring support for these patients. Variations in ventilation modality and time among others can affect patient health [2]. The aim of this study was to clarify the AV situation in national hospitals of El Salvador's public health system.

## Methods

This study is transversal descriptive. A representative sample (*n *= 5) of public national hospitals was selected. These included four second-level hospitals and one third-level hospital. Two hospitals were from the central metropolitan region, one from the par central, west and east region; representing 36.1% of available hospital beds nationwide. To complete AV data of the totality of public hospitals, a telephone survey was used. All data about patients, AV type, costs, maintenance and operative personnel, among others, were collected.

## Results

Only 18% of all public hospitals around the country have mechanical AV. The majority of mechanical AV on the public health network is focused on Rosales National Hospital (HNR), with 61.9% of mechanical ventilators nationwide. Mechanical AV is operated by respiratory therapy personnel at HNR and Zacamil National Hospital; however, in another two hospitals from the east and west region, mechanical AV is operated by residents; and manual AV is provided by self-inflating resuscitator bag operated by interns only. The main causes of AV are nonsurgical; representing 88.9% of the reasons to employ mechanical AV, and 100% for manual AV. The mean patient ventilation time for mechanical AV was >48 hours, and for manual AV was >24 hours. No patient with manual AV had normal pO_2_. The maintenance cost for all mechanical AV was less than $100 per month per ventilator and only 68% of mechanical ventilators were functional, the most frequent model being Servo900.

## Conclusions

The actual mechanical AV existence is very limited, since only four out of 14 departments have this resource, representing 18% of public health centers nationwide, centralized in the department of San Salvador, leaving 82% of these centers with no mechanical ventilation at all, being probably supplanted by manual AV. Results show the necessity for cheaper, efficient and easy-to-use AV systems.
